# BRCAmut and “founder effect”: a prospective study in a single academic institution

**DOI:** 10.18632/oncotarget.24959

**Published:** 2018-04-27

**Authors:** Vera Loizzi, Ettore Cicinelli, Francesco Santamaria, Ferdinando Murgia, Valentina Minicucci, Leonardo Resta, Nicoletta Resta, Maria Iole Natalicchio, Girolamo Ranieri, Gennaro Cormio

**Affiliations:** ^1^ Obstetrics and Gynecology Unit, Department of Biomedical Science and Human Oncology, University of Bari, Bari, Italy; ^2^ Gynecologic Oncology Unit, IRCCS Istituto Oncologico “Giovanni Paolo II”, Bari, Italy; ^3^ Interventional and Medical Oncology Unit, National Cancer Research Center, IRCCS Istituto, Oncologico “Giovanni Paolo II”, Bari, Italy; ^4^ Department of Pathology, University of Bari, Bari, Italy; ^5^ Department of Biomedical Science and Human Oncology, Genetic Unit, University of Bari, Bari, Italy; ^6^ Institute of Molecular Biology Laboratory, Riuniti Hospital, Foggia, Italy

**Keywords:** ovarian cancer, BRCA 1-2

## Abstract

**Introduction:**

About 25% of ovarian cancers can be classified as hereditary. Of these, 80–90% are correleted with the Hereditary Breast–Ovarian Cancer Syndrome (HBOC), which is linked to BRCA 1/2 genes mutations. Our study was set up to study the BRCA-mutation incidence in Apulian population affected with ovarian cancer and to understand the characteristics of the ovarian disease BRCAmut-related.

**Results:**

One hundred and five Apulian patients affected by ovarian cancer with serous high grade histotype, were collected. Of these, 39% were carriers of BRCA 1/2 mutation. BRCAmut patients present a lower median age of onset, a lower percentage of neoplasms in advanced stages and a lower mortality than wild type patients; BRCA-mutated patients have longer mean values of Progression Free Survival (PFS) and Overall Survival (OS).

**Conclusions:**

Apulia is a geographical area with a significant BRCA-mutation incidence variation in the population affected by ovarian cancer. BRCAmut-related ovarian disease is characterized by an earlier median age of onset, an earlier diagnosis and a better outcome than the sporadic disease.

**Materials and Methods:**

From July 2015 to October 2017, all ovarian cancer patients with serous high grade histotype referred to our Institution were prospectly collected. A BRCA-mutation genetic testing after counselling was offered to all of these patients. Clinical characteristics of all ovarian cancer patients were evaluated. Survival curves were estimated by Kaplan-Meier method and compared with log-rank test.

## INTRODUCTION

Ovarian cancer is the second most commonly diagnosed gynaecological cancer worldwide, and the first leading cause of cancer death in females in the western countries [1]. Ovarian malignancies can arise as primary lesions or could be secondary to other neoplasms. About 70% of ovarian tumors originates from the epithelium, while the remaining part starts from the ovarian stromal structures [2]. Ovarian cancer is a silent disease, diagnosed in advanced phase: the clinical onset of the neoplastic disease is characterized by non-specific symptoms (like asthenia, abdominal tension, weight loss). This explains the poor prognosis (only 40% of the patients are still alive after five years from the diagnosis) [3]. Screening strategies in our possession (like pelvic ultrasound, CA-125 levels) are considered poorly effective (UKCTOCS trial [4], ADNEX-MODEL [5], IOTA [6]).

About 25% of ovarian cancers can be classified as hereditary. A small part of these is linked to hereditary syndromes, like Lynch Syndrome (type 2), Peutz-Jeghers, Cowden and Li-Fraumeni syndrome; however about the 80–90% of these hereditary ovarian cancers are connected with the Hereditary Breast–Ovarian Cancer Syndrome (HBOC), which causes 10% of all the breast cancers, 20% of all the ovarian cancers, and is linked to BRCA 1/2 genes mutations [7].

The risk of breast/ovarian cancer in the BRCA-mutation carriers is higher than that of the general population. In addition to the genetic risk, the classical risk factors of ovarian carcinoma including advanced age, nuliparity, endometriosis, obesity and personal or family history of neoplasms increase the risk of ovarian cancer [8].

The BRCA 1/2 genes were discovered on 17q12-21 and 13q12-13 between 1994 and 1995, by the British geneticist M.C. King *et al*. [9], and are mainly involved in DNA repair, but also in the replication fork functioning and cell cycle checkpoints [10].

Besides the HBOC, BRCA 1/2 mutations are responsible for an increased susceptibility to other neoplasms, like prostate cancer, pancreatic cancer, male-breast cancer [11] and also melanoma [12].

A high percentage of BRCA-mutation carriers was found in populations characterized by the so called “founder effect. The founder effect is a phenomenon caused by the so called “inbreeding”, that is, the mating (or breeding) between individuals that are genetically closely related or blood relatives. This could happen in some populations that live isolated, for example due to geographical or religious reasons: by continuous inbreeding, we arrive to the founder effect, based in a partial loss of genetic variability in a new population developed from a small number of individuals [13].

If a pathologic mutation was present in this little number of founders, the expression of deleterious genes will be increased in their descendants. For example this happened in populations like the Ashkenazi Jews (characterized by a high percentage of BRCA 1 mutation carriers) [14] and in the Icelandic people (where an abnormal number of BRCA 2 mutations is found [15].

The aim of our study was to examine the association between the founder effect and the incidence of BRCA-mutation in Apulian population affected with ovarian cancer and to understand the characteristics of BRCAmut-related ovarian cancer.

## RESULTS

### Genetic tests results

One hundred and five Apulian patients affected by ovarian cancer with serous high-grade histotype, were collected. Thirty-nine percent of the patients (*n* = 41) tested positive for BRCA 1/2 mutations.

Of these, BRCA 1 mutation was present in the 71% (*n* = 29) of all the mutated patients whereas the remaining 29% (*n* = 12) were BRCA 2-mutated.

The mutational pattern of the two genes is quite varied, and some mutations are more common than others. The most common mutation is caused by a cytosine insertion (c.5263_6264insC), followed by a substitution (c.65T > C); both these mutations are borne by BRCA1 gene.

c.5263_6264insC constitutes 22% of all the mutations and 31% of BRCA 1 mutations (*n* = 9), while c.65T > C constitutes 12% of all the mutations and 17% of BRCA1 mutations (*n* = 5).

In case of BRCA 2, the most common mutation is a TA-deletion (c.5796_5797delTA), which constitutes 25% (*n* = 3) of BRCA 2-mutations.

### Median age of onset

The median age of the wild type patients was 60 (range, 33, 85) and that of the BRCAmut patients was 53 (range, 35, 78). Particularly, the median age of onset in the BRCA 1-mutated is 51, while in the BRCA 2-mutated is 56.

### Staging and grading at diagnosis

In most cases, the ovarian neoplasm was diagnosed in advanced stage (stage III–IV). Particularly, 78% (*n* = 50) of the neoplasms in wild type patients and 68% (*n* = 28) in BRCAmut patients were diagnosed in advanced stage. The remaining part of ovarian cancers were diagnosed in stages I or II. Regarding the grading, both the sub-groups of patients are characterized by a majority of high-grade tumours (G3). Particularly, 86% (*n* = 55) of tumours in wild type patients are G3 grade, while this percentage is 90% (*n* = 37) in BRCAmut patients sub-group. The remaining cases had a grading G2.

### Mortality rate

Twelve of 105 patients are deceased during the study. Four of these belonged to the sub-group BRCAmut patients (10% of all the mutated patients), while the other 8 were wild type (13% of this sub-group). The cause of death was always linked to the neoplastic disease, while no patients died due to comorbidities or other causes.

### Survival analysis results

**Figure 1:**
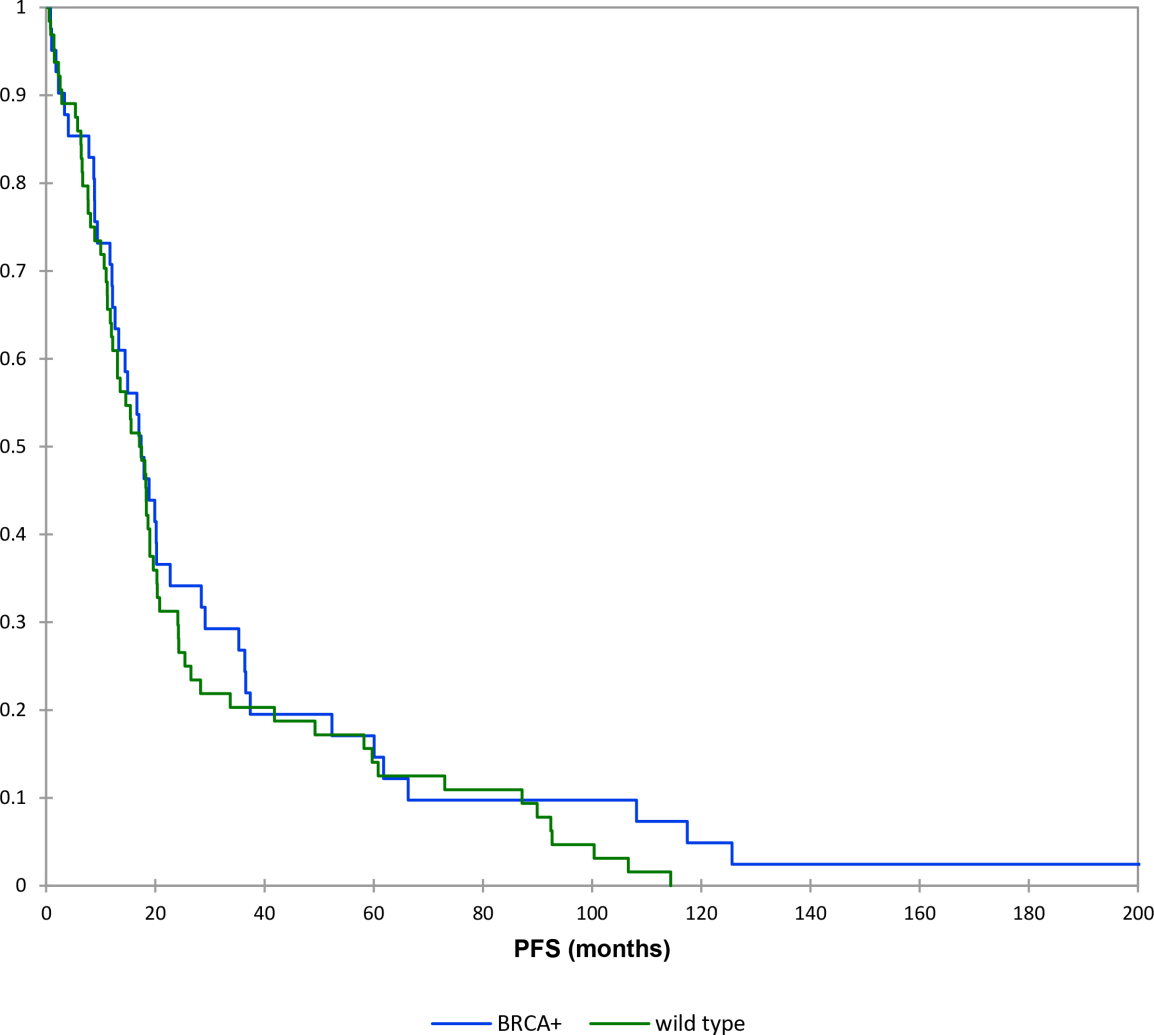
Kaplan–Meier curves: progression free survival (brcamut vs wild type patients)

The mean PFS survival time was 27 months for wild type patients and 34 months for the BRCAmut patients. However, the Kaplan-Meier survival analysis (log-rank test) indicated there was no statistically significant PFS difference between the wild type patients and BRCAmut patients (*P* > 0.05) (Figure 1). BRCAmut patients with neoplasms diagnosed in advanced stages presented a mean PFS value of 30 months, while this value was 21 months in wild type patients with the same features. However, the test of equality (log-rank test) considered this difference as non-significant (*p value* > 0.05).

**Figure 2:**
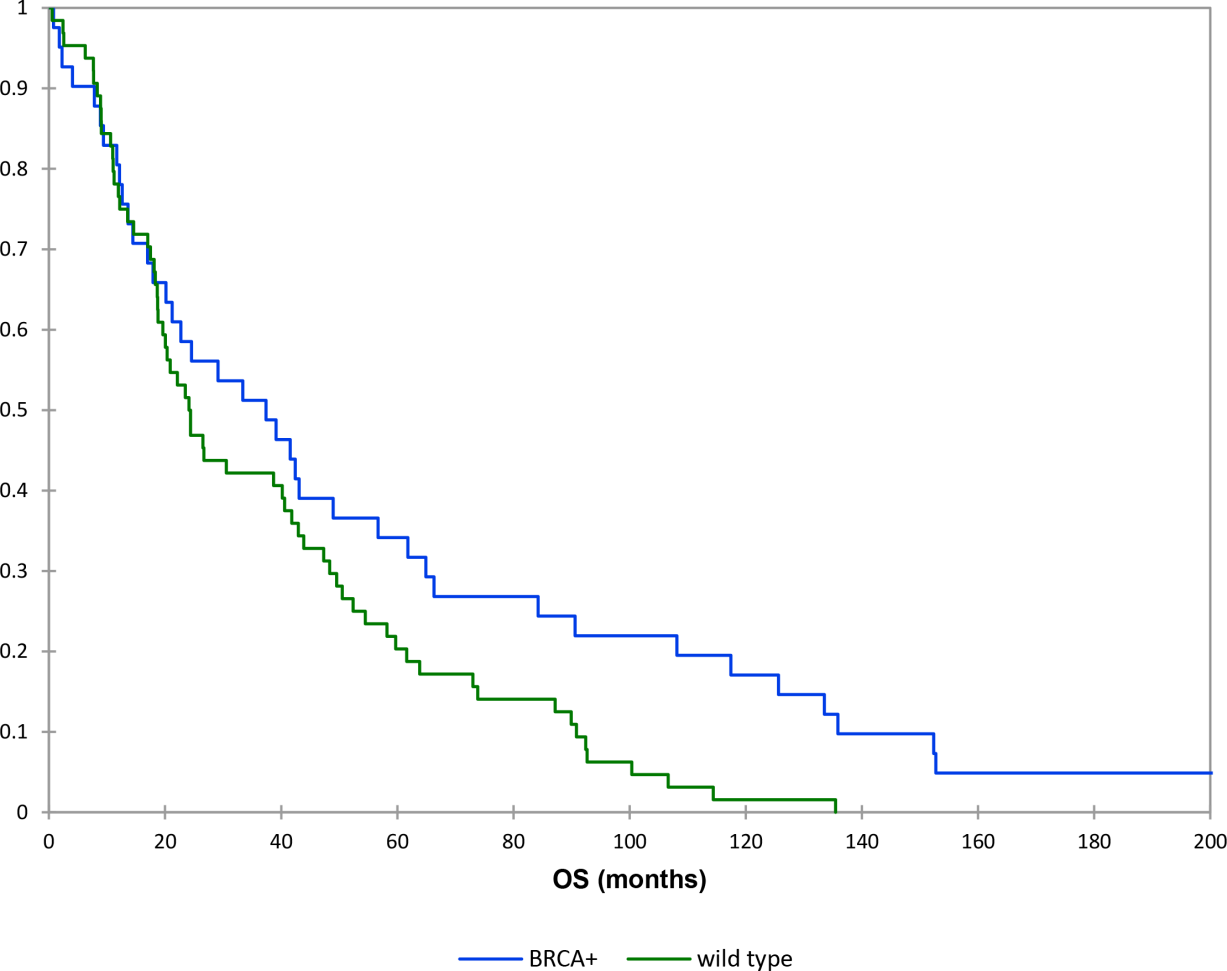
Kaplan–Meier curves: overall survival (brcamut vs wild type patients)

The mean OS time for the wild type patients was 38 months compared to 87 months in BRCAmut patients. The Kaplan-Meier survival analysis indicated there was statistically significant difference in OS between the two groups (*P* = 0.03) (Figure 2).

The mean OS time of BRCAmut ovarian cancer patients with advanced stage was 102 months, while that of the wild type ovarian cancer patients with advanced stage was 34 months (*P* = 0.04).

In the same way, the results about overall survival in high grade tumors showed a mean OS time of 87 months for BRCAmut patients versus 34 months for wild type patients. This showed a positive trend however this difference was considered non-significant by test of equality (*p value* > 0.05) probably due to the necessity of expanding the sample group.

To examine the variables identified as important in univariate analyses further, a multivariate analysis was performed. BRCA mutation and stage remained as a significant independent poor prognostic factor for survival in our patient population (Table 1).

**Table 1:** Multivariate analysis of survival based on clinical and pathologic factors

Variables	Hazard ratio	*P*-value
Advanced stage (IIB–IV)	0.59	0.04
BRCA mutation	0.62	0.03

## DISCUSSION AND CONCLUSIONS

Results of our study showed that Apulia is a region with a significant BRCA-mutation incidence in the population affected by ovarian cancer, since we observed an incidence of 39%, that is larger than that typically observed in the general Italian population. In fact in Italy, 4–20% of the identified mutations recurred among apparently unrelated families, and significant regional founder effect has been demonstrated for few mutations [16–17]. More or less elevated variations of this percentage are known in medical literature: they are linked to particular geographical areas, such as Israel (32%), Sweden (8%), Fiji Islands (17%), Florida (16%). Another important cause of incidence variation is the so-called “inbreeding” that leads to the phenomenon known as “founder effect”.

We identified a total of 41 *BRCA1* and *BRCA2* mutations. The c.5263_6264insC mutation accounts for 31% and c.65T > C mutation for 17% of families with BRCA1 mutations. On the other hand we found that c.5796_5797delTA mutation accounts for 25% of the BRCA2 positive families. From our results, the three mutations appear to be an unique founder mutations in the Apulia region.

Data from our study showed that BRCA mutated ovarian cancer patients were diagnosed at a mean age of 53 years; these ages are less than the average age at onset of wild type ovarian cancer which showed to be of 60 years. More specifically, BRCA1 mutated patients had a median age of onset of 51 whereas BRCA 2-mutated patients had a median age of onset of 56 years. Our study also revealed that the majority of BRCA mutated patients had G3 disease and are diagnosed in an earlier stage. Moreover, we found a better outcome in terms of overall survival for BRCA mutated ovarian cancer patients compared to the wild type women. In fact the overall survival for the mutated group was 87 compared to 38 months for the control group. These differences were also showed after stratified for stage and grade. On the other hand, the progression free survival was not different between the two groups and this probably due to the necessity of expanding the sample group.

Our results showing a better outcome in terms of overall survival for BRCA mutated ovarian patients confirmed what has been recently published [18]. This may be explained based on several factors such as the major response to the platinum compounds observed for BRCA mutated patients and in addition the sensibility of this disease to the targeted-therapy based on PARP-inhibitors (such as olaparib). On the other hand, for other tumors this correlation between BRCA mutation and favorable prognosis has not been observed. In fact, some authors described a worse prognosis in breast cancer patients which are mutated in BRCA gene [19, 20].

The identification of *BRCA1* and *BRCA2* mutation carriers and the individualized risk assessment is an important procedure that is growing in clinical setting since management protocols for mutation carriers become well established and proven life-saving, risk-reducing preventive medical interventions exist. When a mutation is identified in a family, an oncogenetic test with predictive information should be presented to all the members of the family. For this reason, oncogenetic testing is showing to be a strong therapeutical predictive tool, as new target therapy, such as PARP inhibitors emerge and chemosensitivity to platinum based therapy is documented.

Knowledge about mutation distibution diversity is important not only for the consideration of country specific cost-efficient strategy for mutation screening, but also for the ovarian cancer control and prevention through more liberal, yet rational, genetic testing and counseling in a globalized landscape of postmodern Europe. The identification of individuals with high risks will be useful for the early-detection of the neoplastic disease. Therefore, we hope that the early-detection of the tumor associated with the increasing role of targeted-therapy such as PARP-inhibitors will give more chance of cure to women affected by ovarian cancer.

## MATERIALS AND METHODS

### Study participants

#### Inclusion criteria

1. Female subjects willing to sign the informed consent form (ICF);
2. Subjects who are at least 18 years of age at the time of informed consent;
3. Patients with a histologically confirmed diagnosis of serous epithelial ovarian cancer including primar peritoneal and fallopian tube malignances;
4. Patients with origin from Apulia (or immediate surroundings).

#### Exlusion criteria

1. Patients with non-epithelial origin of the ovary, the fallopian tube or the peritoneum;
2. Patients with ovarian tumors of low malignant potential (e.g. borderline tumors), or mucinous carcinoma
3. Patients with other malignancy within the last 5 years

After genetic counselling and informed consent, carried out between 15th July 2015 and 4th October 2017 all the patients were subjected to genetic test, through Sanger method + NGS on EDTA-blood, so as to find out the BRCA 1/2 genes mutational status. Almost all the tests were carried out by the U.O.C. Medical Genetics Lab – A.O.U. Policlinico di Bari (Italy), while a few tests were executed in other Italian places. After the oncological treatment, all the patients were followed-up at our clinic.

### Exposure

The term “founder” is used for those mutations where haplotype studies revealed shared polymorphic markers consistent with common ancestor, or when unrelated mutation carriers were repeatedly identified (at least 3 times). Some mutations previously described as founder mutations in one country, subsequently are found at a higher proportion in other countries/regions as true founders. These mutations in adjacent countries will likely reflect the gradient transition from the “epicenter” over the time due to historical co-existence of different populations in the same region.

### Outcome

#### Our study was characterized by different endpoints

first of all, our aim was set to discover BRCA-mutation carriers incidence in our population of Apulian patients affected with ovarian cancer, in order to compare it with the general population incidence (20%).

Then, we examined the mutations epidemiology, and examined the traits of the ovarian cancer including median age of onset, staging and grading at diagnosis and mortality rate, comparing the BRCA mutation-related cancer to the sporadic disease.

1. At last, we conducted survival analysis (PFS and OS) of the two sub-groups of ovarian cancer patients (BRCA+ and wild type), so as to compare the survival probability (through the Kaplan-Meier estimator) and to find out potential differences in the prognosis of the two sub-types of ovarian neoplastic disease. The analysis of the survival functions was then carried on only considering particular sub-categories, such as patients diagnosed in an advanced state or patients with high-grade neoplasms.

### Covariates

The advanced stage of the neoplastic disease and the mutational status of BRCA pattern genes (wild type/BRCAmut) are the variables (covariates) involved in the possible influence over the survival of the group of patients under investigation.

### Statistical analysis

The software used for the survival analysis was XLSTAT^®^. Kaplan-Meier analyses were used to determine OS and PFS, starting from the end of first-line chemotherapy to the date of last follow-up. Differences between the two groups (BRCA muteted and wild typre ovarian cancer patients) in OS and PFS were tested by log-rank tests. All tests with *p* values less than 0.05 were considered significant. Multivariate analyses were performed with the use of Cox proportional hazards regression. A two-sided probability value less of 0.05 was considered to be significant.

## References

[1] Permuth-Wey J, Sellers TA (2009). Epidemiology of ovarian cancer. Methods Mol Biol.

[2] Kurman RJ, Shih IeM (2010). The origin and pathogenesis of epithelial ovarian cancer: a proposed unifying theory. Am J Surg Pathol.

[3] Goff BA, Mandel LS, Melancon CH (2004). Frequency of symptoms of ovarian cancer in women presenting to primary care clinics. JAMA.

[4] Menon U, Gentry-Maharaj A, Hallett R, Ryan A, Burnell M, Sharma A, Lewis S, Davies S, Philpott S, Lopes A, Godfrey K, Oram D, Herod J (2009). Sensitivity and specificity of multimodal and ultrasound screening for ovarian cancer, and stage distribution of detected cancers: results of the prevalence screen of the UK Collaborative Trial of Ovarian Cancer Screening (UKCTOCS). Lancet Oncol.

[5] International Ovarian Tumor Analysis Group (2014). IOTA Welcome page.

[6] Timmerman D, Ameye L, Fischerova D, Epstein E, Melis GB, Guerriero S, Van Holsbeke C, Savelli L, Fruscio R, Lissoni AA, Testa AC, Veldman J, Vergote I (2010). Simple ultrasound rules to distinguish between benign and malignant adnexal masses before surgery: prospective validation by IOTA group. BMJ.

[7] Lynch HT, Casey MJ, Snyder CL, Bewtra C, Lynch JF, Butts M, Godwin AK (2009). Hereditary ovarian carcinoma: heterogeneity molecular genetics, pathology and management. Molecular Oncology.

[8] Fortner RT, Ose J, Merritt MA, Schock H, Tjønneland A, Hansen L, Overvad K, Dossus L, Clavel-Chapelon F, Baglietto L, Boeing H, Trichopoulou A, Benetou V (2015). Reproductive and hormone-related risk factors for epithelial ovarian cancer by histologic pathways, invasiveness and histologic subtypes: Results from the EPIC cohort. Int J Cancer.

[9] Ford D, Easton DF, Bishop DT, Narod SA, Goldgar GE (1994). Risks of cancer in BRCA1-mutation carriers. Lancet.

[10] Roy R, Chun J, Powell SN (2002). Cancer Susceptibility and the Functions of BRCA1 and BRCA2. Cell.

[11] Liede A, Karlan BY, Narod SA (2004). Cancer Risks for Male Carriers of Germline Mutations in BRCA1 or BRCA2: A Review of the Literature. J Clin Oncol.

[12] Cruz C, Teule A, Caminal JM, Blanco I, Piulats JM (2011). Uveal Melanoma and BRCA1/BRCA2 Genes: A Relationship That Needs Further Investigation. J Clin Oncol.

[13] Drayna D (2004). Founder Mutations - A special class of genetic mutations that often cause human disease is enabling scientists to trace the migration and growth of specific human populations over thousands of years. Sci Am.

[14] Anglian Breast Cancer Study Group (2000). Prevalence and penetrance of BRCA1 and BRCA2 mutations in a population-based series of breast cancer cases. Br J Cancer.

[15] Thorlacius S, Olafsdottir G, Tryggvadottir L, Neuhausen S, Jonasson JG, Tavtigian SV, Tulinius H, Ogmundsdottir HM, Eyfjörd JE (1996). A single BRCA2 mutation in male and female breast cancer families from Iceland with varied cancer phenotypes. Nat Genet.

[16] Nedelcu R, Liede A, Aubé J, Finch A, Kwan E, Jack E, Narod SA, Randall S, Hugel L, Clark K (2002). BRCA mutations in Italian breast/ovarian cancer families. Eur J Hum Genet.

[17] Farghaly SA (2000). Current status of management of hereditary ovarian–breast cancer syndrome. Am J Obst Gyn.

[18] Vencken PM, Kriege M, Hoogwerf D, Beugelink S, van der Burg ME, Hooning MJ, Berns EM, Jager A, Collée M, Burger CW, Seynaeve C (2011). Chemosensitivity and outcome of BRCA1- and BRCA2-associated ovarian cancer patients after first-line chemotherapy compared with sporadic ovarian cancer patients. Ann Oncol.

[19] Zhu Y, Wu J, Zhang C, Sun S, Zhang J, Liu W, Huang J, Zhang Z (2016). BRCA mutations and survival in breast cancer: an updated systematic review and meta-analysis. Oncotarget.

[20] Xie Y, Gou Q, Wang Q, Zhong X, Zheng H (2017). The role of BRCA status on prognosis in patients with triple-negative breast cancer. Oncotarget.

